# Medical Students and Clinicians’ Perceptions of Social Media Direct-to-Consumer Advertising and Medication Requests

**DOI:** 10.2196/91024

**Published:** 2026-04-23

**Authors:** Erin Willis, Samuel M Tham, Janelle Applequist, Delaney Fouts, Ayra Ali, Charles Mulkey, Natali Shaver

**Affiliations:** 1 Advertising, Public Relations, and Design, University of Colorado Boulder, 1511 University Ave, 478 UCB, Boulder, CO, 80309, United States, 1 4175931005; 2 Department of Journalism and Media Communication, Colorado State University, Fort Collins, CO, United States; 3 Zimmerman School of Advertising and Mass Communication, University of South Florida, Tampa, FL, United States; 4 School of Medicine, University of Colorado Anschutz Medical Campus, Aurora, CO, United States; 5 School of Medicine, University of Missouri–Kansas City, Kansas City, MO, United States

**Keywords:** pharmaceutical advertising, social media, telehealth, GLP-1 agonists, glucagon-like peptide-1

## Abstract

This study examines how medical students and clinicians report experiencing patient medication requests associated with prescription drug direct-to-consumer advertising on social media; survey data from 98 respondents indicate that those providing both in-person and virtual care encounter more frequent requests for medications advertised online, particularly branded glucagon-like peptide-1 (GLP-1) weight loss drugs.

## Introduction

Digital media have become important to pharmaceutical marketing, transforming both the scale and tone of direct-to-consumer advertising (DTCA). A 2024 industry analysis suggested that pharmaceutical brands accounted for approximately 88% of digital advertising spending in the health care sector [1]. Emerging studies show that social media DTCA may influence patient expectations [2], which may contribute to requests for specific medications during clinical visits [3].

Robinson et al [4] conducted one of the few physician investigations of DTCA, finding that 88% of primary care physicians believed DTCA prompted patients to request specific medications, with 65% agreeing it increased time spent with patients. Two decades later, comparable studies remain scarce, likely due to the inherent challenges of accessing clinicians for research [5,6]. This study examines how medical students and clinicians perceive medication requests associated with social media DTCA across in-person and virtual care settings.

## Methods

### Ethical Considerations

The University of Colorado Boulder Institutional Review Board approved the study and granted a waiver of signed informed consent due to minimal risk and anonymous data collection (protocol number 25‐0076). Participants were provided with an electronic consent statement prior to beginning the survey and indicated consent by proceeding. Participation was voluntary, and respondents could exit the survey at any time without penalty. As compensation, participants were entered into a raffle to win one of fifteen US $50 gift cards.

### Survey Instrument

A cross-sectional online survey was distributed using snowball sampling among medical students and clinicians in Colorado and Missouri. Ninety-eight valid responses were included in the analysis: medical students (n=85), residents (n=5), physicians (n=6), and nurse practitioners (n=2). Respondents evaluated items measuring clinical practice type (in-person, virtual), attitudes toward DTCA, and patient medication requests (eg, glucagon-like peptide-1 [GLP-1], Wegovy). All survey items were measured using 5-point Likert-type scales. Survey items assessing DTCA attitudes were adapted from Robinson et al [4] for relevance to students and clinicians in digital contexts; formal validation in student populations has not been established. Medication request items were developed to assess the frequency of patient requests for specific drug categories commonly promoted on social media [7]. K-means clustering grouped respondents by reported clinical setting (in-person only vs in-person and virtual); clustering was based on self-reported frequency of clinical practice in virtual and in-person settings. Multiple linear regression examined whether DTCA attitude items predicted the frequency of self-reported medication requests associated with social media advertising.

## Results

Two clusters emerged: (1) in-person only and (2) in-person and virtual respondents (Table 1). The hybrid group reported significantly more patient requests for medications seen on social media (mean 3.15, SD 0.79) than the in-person group (mean 2.64, SD 0.91; *F*_1,96_=8.67, *P*=.006). The effect size was medium (*η*^2^=0.085). Requests were highest for GLP-1 agonists (eg, semaglutide) and branded weight loss medications (eg, Wegovy, Zepbound), with similar differences observed for sodium-glucose cotransporter-2 (SGLT-2) inhibitors. Given the exploratory nature of the study, adjustments for multiple comparisons were not applied.

**Table 1.** Patient requests for specific medications by practice type.^a^

Medication category	In-person mean (SD)	Hybrid mean (SD)	*P* value^b^
GLP-1 (glucagon-like peptide-1) agonists (eg, semaglutide)	3.18 (1.21)	3.89 (0.87)	*P*=.001
Wegovy	2.33 (1.21)	3.26 (1.02)	*P*<.001
Zepbound	2.11 (1.25)	2.83 (1.17)	*P*=.004
SGLT-2 (sodium-glucose cotransporter-2 ) inhibitors	2.04 (0.97)	2.53 (0.87)	*P*=.011
Metformin	1.96 (0.93)	2.26 (0.90)	*P*=.099
Compounded semaglutide	2.34 (1.24)	2.77 (1.14)	*P*=.76

a Means reflect Likert scale responses.

b Two-group comparisons from one-way ANOVA.

In regression analyses, respondents who perceived that DTCA influences prescribing practices (*β* =.24, *P*=.035) and increases patient requests for specific medications (*β*=.35, *P*=.012) reported higher frequencies of medication requests associated with social media advertising. The overall model accounted for 27% of variance (R^2^=.27).

## Discussion

### DTCA in Practice

These findings align with previous assertions that digital DTCA may influence medication requests, particularly in virtual settings [3]. DTCA may be associated with patients expressing preferences for branded medications prior to visits. Previsit preference formation may shape clinical discussions, particularly in shorter virtual visits [8,9].

DTCA on social media may influence patients’ expectations entering clinical visits, potentially increasing the salience of DTCA prior to medical evaluation. The notable influence of GLP-1 drug promotions illustrates how digital DTCA may be associated with heightened visibility of specific drug classes online. Because the sample consisted primarily of medical students, these findings should be interpreted as reflecting student and early-career perceptions rather than established prescribing behavior. Treatment deliberation may increasingly be informed by online content. However, these interpretations are exploratory and warrant further investigation in samples of actively practicing clinicians.

### Clinical and Public Health Implications

As digital pharmaceutical promotions become more ubiquitous, medical students and clinicians may benefit from structured approaches for navigating patient requests shaped by online content. Skills in digital health literacy and expectation management may become core competencies, particularly in virtual visits where time is constrained.

From a public health perspective, social media DTCA may also widen disparities in medication access and understanding. Algorithms differentially target content based on demographic and behavioral data, meaning that some patients may be disproportionately exposed to GLP-1 promotions while others remain unaware of treatment alternatives. Integrating digital health literacy into preventive care could help mitigate these inequities and support more informed patient expectations.

### Limitations

Physician surveys are known to have comparatively low response rates in general, which means that even achieving small samples represents substantial field access and resource cost. The predominance of medical students in this sample limits the ability to generalize findings to actively prescribing clinicians. Findings should therefore be interpreted as exploratory.

### Conclusion

Digital DTCA is increasingly visible in the informational environment that often precedes clinical visits. In this sample, respondents reporting clinical practice in both in-person and virtual settings described significantly more frequent patient requests for GLP-1 and other branded medications associated with social media DTCA. These patterns suggest that digital advertising may shape patient expectations entering clinical settings, particularly in virtual contexts. Given the primarily medical student sample, conclusions regarding prescribing behavior should be interpreted in that light. Clinicians will need skills in digital health literacy and expectation management to preserve evidence-based prescribing while communicating respectfully about drugs that patients discover online. Future research should examine how training programs and regulatory bodies can better equip clinicians for a landscape where social media platforms serve as de facto medication counselors.

## Abbreviations

DTCA: direct-to-consumer advertising
GLP-1: glucagon-like peptide-1
SGLT-2: sodium-glucose cotransporter-2 inhibitor
